# Clinical Utility of Comprehensive Genomic Profiling in a Community Hospital Outside the Cancer Genomic Core Hospital Network: A Single-Center Retrospective Cohort Study

**DOI:** 10.7759/cureus.101510

**Published:** 2026-01-14

**Authors:** Akinori Sasaki, Rika Kimura

**Affiliations:** 1 Gastroenterology, Tokyo Bay Urayasu Ichikawa Medical Center, Urayasu, JPN

**Keywords:** actionable genomic alteration, community hospital outside the cancer genomic core hospital network, comprehensive genomic profiling, druggable genomic alteration, genomically matched therapy

## Abstract

Background

Comprehensive genomic profiling (CGP) has become increasingly integrated into precision oncology; however, its real-world clinical utility in community hospitals outside the national cancer genomic core hospital network in Japan remains less studied. This study aimed to evaluate the implementation, feasibility, and clinical impact of CGP in a community-based hospital.

Methods

We retrospectively reviewed patients with unresectable or recurrent solid tumors who had not received systemic chemotherapy at our hospital between April 2021 and December 2025. Clinical outcomes, including the detection rate of druggable genomic alterations, the proportion of patients who received genomically matched therapy, and overall survival (OS), were compared between patients who underwent CGP and those who did not.

Results

Among 253 patients, 60 (24%) underwent CGP testing. Druggable genomic alterations were identified in 45 patients (75%), and seven patients (12%) received genomically matched therapy. Of these, 5% were treated within clinical trials, and 7% received approved targeted agents. Among patients who received matched therapy, the best overall response was complete response (CR) in two, partial response (PR) in two, stable disease (SD) in one, and progressive disease (PD) in two. Tumor-type-stratified analyses showed variability in actionable/druggable profiles and matched-therapy delivery across tumor types. No significant difference in OS was observed between the CGP and non-CGP groups (median OS: 22.9 vs. 23.0 months, P = 0.78). Within major tumor types, including colorectal, gastric, pancreatic, and biliary tract cancers, OS did not significantly differ according to CGP testing status. Among CGP patients, OS tended to be longer in those who received matched therapy, although the difference was not statistically significant.

Conclusions

Despite being conducted in a community hospital outside the cancer genomic core network, CGP testing was feasible and enabled a clinically meaningful proportion of patients to access genome-matched therapy at rates comparable to those reported from tertiary centers. Although CGP did not directly translate into improved OS, it provided valuable treatment opportunities and facilitated precision oncology in a regional care setting. Further expansion of accessible genome-guided therapies may enhance the clinical impact of CGP in community hospitals.

## Introduction

Improving overall survival (OS) and quality of life (QoL) for patients with cancer increasingly relies on personalized treatment. Comprehensive genomic profiling (CGP) based on next-generation sequencing (NGS) has been widely implemented across tumor types and now underpins biomarker-driven therapy selection and clinical trial enrollment [1,2]. In Japan, CGP has been reimbursed since 2019 primarily for patients with unresectable or recurrent solid tumors who have completed (or are expected to complete) standard systemic therapies, and for selected malignancies with limited established standard options (e.g., rare cancers or cancers of unknown primary). As of 2025, multiple CGP tests, including blood-based assays, are available under public insurance [3,4]. Prior studies further suggest that patients who receive matched therapies guided by actionability identified through CGP derive survival benefits [5,6].

However, much of the existing evidence originates from high-volume cancer centers and university hospitals, particularly designated core cancer genomic medicine hospitals [3,7,8]. In community hospitals, CGP often cannot be completed in-house, necessitating specimen submission to external laboratories and the use of external molecular tumor boards (MTBs); moreover, access to matched therapies or clinical trials typically requires referral to tertiary centers. Patients managed in community settings are also more likely to be older and to have poorer performance status (PS), and they face additional implementation barriers such as travel distance and transfer burden. Accordingly, it is clinically important to determine whether performing CGP in the community context truly translates into meaningful patient benefit, especially in terms of survival.

Our institution is a general community hospital rather than a designated genomic medicine center, yet we have actively implemented CGP in routine practice. Using a consecutive real-world cohort of patients with solid tumors treated at our hospital, we aim to evaluate the association between CGP implementation and survival outcomes while concurrently assessing key implementation indicators, including submission rate, recommendation acceptance rate, time-to-report and time-to-action (turnaround time (TAT)), and rates of treatment/trial access, to clarify the utility of CGP in community hospitals.

## Materials and methods

Study population

This retrospective study included patients with solid tumors who received systemic chemotherapy in the Department of Medical Oncology at Tokyo Bay Urayasu Ichikawa Medical Center between April 2021 and December 2025. Eligible patients had a diagnosis of unresectable or recurrent solid cancer and had received at least one line of chemotherapy. This study focused on unresectable or recurrent disease to reflect the real-world setting in which CGP is typically considered and reimbursed in Japan, namely, patients with advanced solid tumors who have limited standard treatment options. Informed consent was obtained by an opt-out procedure; patients who declined the use of their medical information were excluded. All data were de-identified prior to analysis, and each patient was assigned a study-specific anonymized identifier; no directly identifiable personal information was accessible to the investigators during analysis. The study protocol was approved by the Institutional Review Board of Tokyo Bay Urayasu Ichikawa Medical Center (approval no. 1141) and the study was conducted in accordance with the Declaration of Helsinki.

Characteristics of our hospital

Our institution is a community-based general hospital located in Urayasu, Chiba, Japan. Although we have a Department of Medical Oncology, we are neither a designated core hospital for cancer genomic medicine nor a cooperative (affiliated) hospital within the national program. For CGP, patients are referred to Juntendo University Urayasu Hospital, a designated cooperative hospital for cancer genomic medicine, located approximately 5 km from our facility. When CGP identifies eligibility for a clinical trial, patients are referred to the National Cancer Center Hospital East, a designated core hospital for cancer genomic medicine, located about 35 km from our hospital.

Next-generation sequencing-based comprehensive genomic profiling tests

During the study period, tissue-based CGP was performed using FoundationOne® CDx Cancer Genomic Profile, OncoGuide™ NCC Oncopanel System, and GenMineTOP. In contrast, blood-based CGP was conducted using FoundationOne® Liquid CDx Cancer Genomic Profile and Guardant360 CDx. When adequate tumor tissue was available, a tissue-based CGP test was preferentially performed; the specific test kit to be used was determined by the referral/designated cancer genomic medicine hospital.

In this study, “actionable genomic alterations” for treatment selection were defined as those predicted to confer sensitivity or resistance to either approved standard-of-care agents or investigational targeted therapies in clinical trials [9]. Potentially targetable alterations included not only gene mutations but also high microsatellite instability (MSI-high) and high tumor mutational burden (TMB), defined as ≥10 mutations per megabase. “Druggable genomic alterations” were defined as genomic abnormalities judged by the MTB to be therapeutically targetable, for which treatment with an approved drug, investigational new drug (IND), or off-label agent was considered feasible (7). “Genomically matched therapy” was defined as any treatment actually administered on the basis of the patient’s CGP results [10].

Assessment

The primary objective of this study was to evaluate the current status and clinical utility of CGP in a community-based hospital. We investigated the implementation rate of CGP among patients with unresectable solid tumors who had received at least one line of systemic chemotherapy at our institution. We also assessed the proportion of patients harboring druggable genomic alterations and the proportion who actually received genomically matched therapy based on these alterations. In addition, we evaluated and compared the OS between patients who underwent CGP testing and those who did not in the entire cohort. Among patients who received CGP, OS was also compared between those who received genomically matched therapy and those who did not.

Statistical analysis

Statistical comparisons of baseline characteristics between the CGP and non-CGP groups were performed using the χ² test or Fisher’s exact test for categorical variables and Student’s t-test or the Mann-Whitney U test for continuous variables, as appropriate. OS was defined as the interval from the initiation of first-line chemotherapy to death from any cause; patients alive at the last follow-up were censored at that date. OS was estimated and displayed using the Kaplan-Meier method, and survival rates with 95% confidence intervals (CI) were reported at prespecified time points. Survival curves were compared with the log-rank test. Hazard ratios (HR) and 95% CIs for the CGP versus non-CGP comparison were estimated using the Cox proportional hazards model. A multivariable Cox model was fitted to adjust the HR for clinical covariates that differed between groups. All statistical analyses used a two-sided α of 0.05 and were conducted with SPSS Statistics (version 29, IBM Corp., Armonk, NY).

## Results

Between April 2021 and December 2025, 253 patients with malignant tumors received palliative systemic chemotherapy in the Department of Medical Oncology at our hospital. Among them, 60 patients underwent CGP testing. Two additional patients underwent CGP testing but did not receive systemic chemotherapy; these two patients were excluded from the present study. Baseline characteristics of patients in the CGP and non-CGP groups are summarized in Table 1. The CGP group comprised 60 patients, whereas the non-CGP group comprised 193 patients. Median age was younger in the CGP group than in the non-CGP group (64.5 vs. 73.0 years, P < 0.001). ECOG PS also differed between the groups: PS 0 was more frequent in the CGP group than in the non-CGP group (30 (50.0%) vs. 53 (27.5%), P < 0.001), whereas PS 1-2 was more common in the non-CGP group (137 (71.0%) vs. 30 (50.0%), P < 0.001).

**Table 1:** Patient characteristics in the overall population. Data are presented as median (range) or n (%). Differences in continuous variables were assessed using the Student’s t-test (two-sided). Categorical variables were compared using Pearson’s chi-square test. For variables with multiple categories (e.g., ECOG PS and cancer types), an overall chi-square test was applied. A P-value < 0.05 was considered statistically significant. CGP, comprehensive genomic profiling; ECOG PS, Eastern Cooperative Oncology Group performance status; GIST, gastrointestinal stromal tumor; NET, neuroendocrine carcinoma; NSCLC, non-small cell lung cancer; SCLC, small cell lung cancer

Features	CGP group (n = 60)	Non-CGP group (n = 193)	Test statistic	P-value
Age, years				
Median (min-max)	64.5 (39-88)	73.0 (33-89)	U = 3860	<0.001
Male, n (%)	39 (65.0)	125 (64.8)	χ² = 0.002	0.97
ECOG PS, n (%)				
0	30 (50.0)	53 (27.5)	χ² = 22.8	<0.001
1	26 (43.3)	83 (43.0)		
2	4 (6.7)	54 (28.0)		
3	0 (0)	3 (1.6)		
Cancer types				
Colorectal cancer	17 (28.3)	64 (33.2)	χ² = 62.64	<0.001
Gastric cancer	5 (8.3)	33 (17.1)		
Pancreatic cancer	11 (18.3)	20 (10.4)		
Breast cancer	3 (5.0)	18 (9.3)		
Biliary tract cancer	18 (30.0)	5 (2.6)		
Hepatocellular carcinoma	0 (0)	21 (10.9)		
Ovarian cancer	2 (3.3)	6 (3.1)		
Prostate cancer	0 (0)	6 (3.1)		
Esophageal cancer	1 (1.7)	5 (2.6)		
Endometrial cancer	1 (1.7)	3 (1.6)		
NSCLC	0 (0)	4 (2.1)		
Renal cell carcinoma	0 (0)	3 (1.6)		
GIST	0 (0)	2 (1.0)		
NET	0 (0)	2 (1.0)		
Duodenal cancer	1 (1.7)	0 (0)		
Retroperitoneal liposarcoma	1 (1.7)	0 (0)		
SCLC	0 (0)	1 (0.5)		
Organs with metastases, n (%)				
≤2	51 (85.0)	166 (86.0)	χ² = 0.04	0.85
≥3	9 (15.0)	27 (14.0)		
Site of metastases, n ( %)				
Lymph node	43 (71.7)	133 (68.9)	χ² = 0.16	0.69
Liver	33 (55.0)	82 (42.5)	χ² = 2.89	0.089
Lung	9 (15.0)	44 (22.8)	χ² = 1.68	0.19
Peritoneum	17 (28.3)	44 (22.8)	χ² = 0.77	0.38
Bone	6 (10.0)	22 (11.4)	χ² = 0.09	0.76
Comorbidity				
Hypertension	28 (46.7)	97 (50.3)	χ² = 0.24	0.63
Diabetes mellitus	13 (21.7)	42 (21.8)	χ² = 0.00	0.99
Cardiac disease	5 (8.3)	43 (22.3)	χ² = 5.79	0.016
Lung disease	3 (5.0)	17 (8.8)	χ² = 0.91	0.34
Renal disease	4 (6.7)	19 (9.8)	χ² = 0.56	0.46

**Figure 1:**
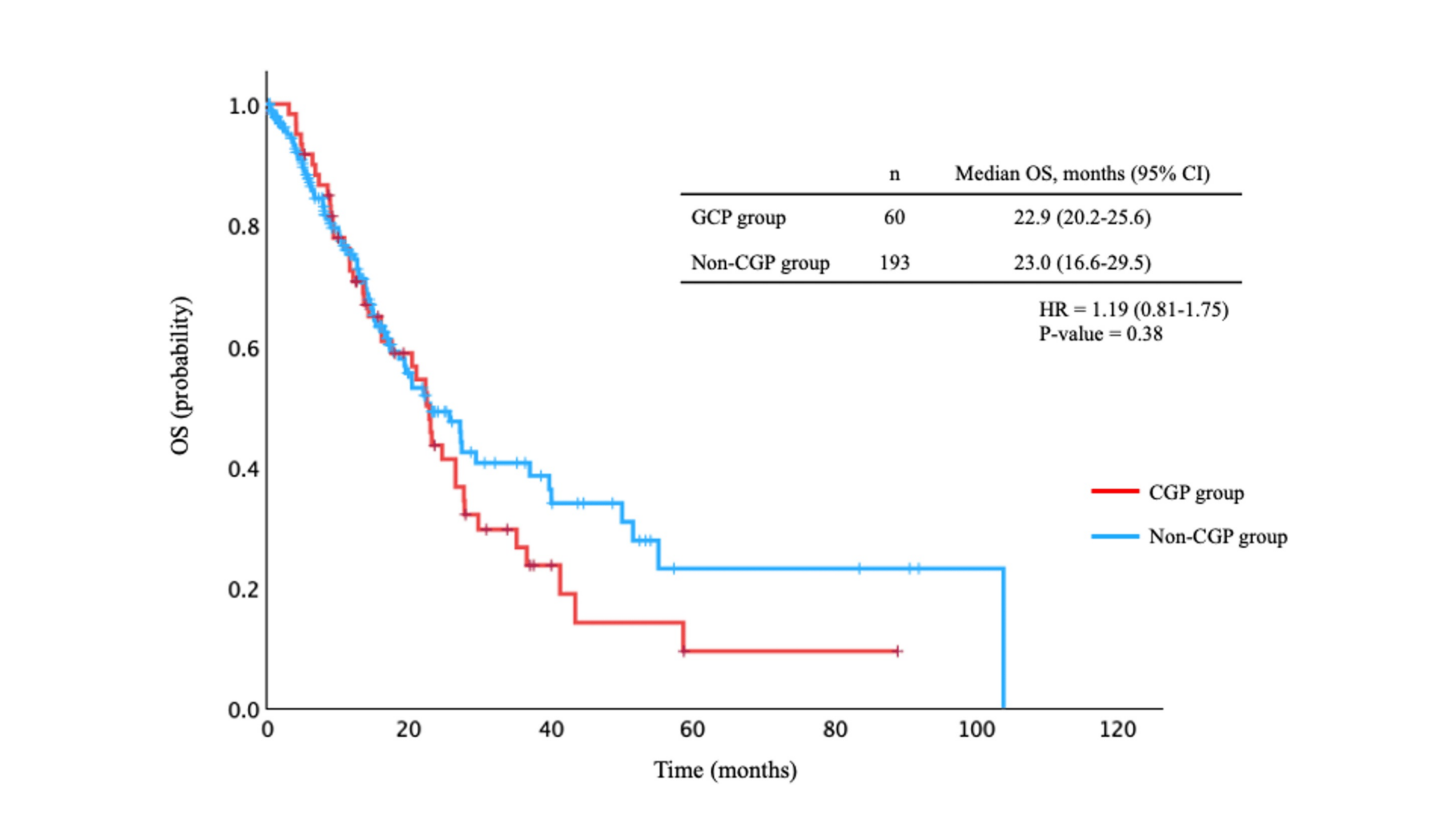
Overall survival (OS) curves in the comprehensive genomic profiling (CGP) and non-CGP groups. Figure 1 shows OS in the CGP and non-CGP groups. No significant difference in OS was observed between the two groups (median OS: 22.9 months in the CGP group vs. 23.0 months in the non-CGP group, P = 0.78).

**Figure 2:**
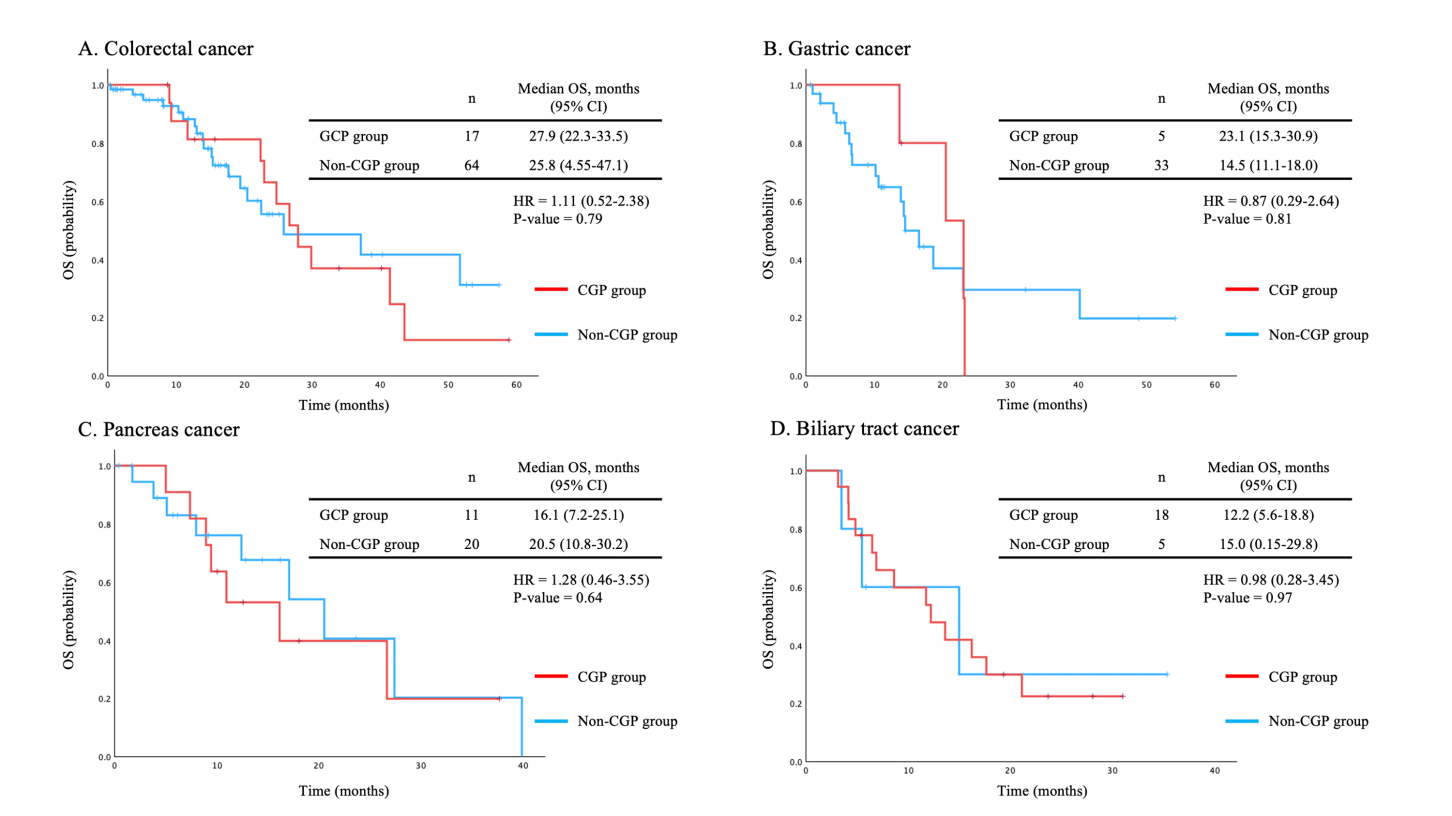
Overall survival (OS) according to the presence or absence of comprehensive genomic profiling (CGP) testing within major tumor types, including (A) colorectal, (B) gastric, (C) pancreatic, and (D) biliary tract cancers. Figure 2 shows overall survival according to the presence or absence of CGP testing within major tumor types, including (A) colorectal, (B) gastric, (C) pancreatic, and (D) biliary tract cancers. There was no statistically significant difference in OS across any of the cancer types.

The distribution of primary tumor sites also differed between the groups. In the CGP group, biliary tract cancer was the most frequent tumor type, accounting for 18 (30.0%) cases. In contrast, hepatocellular carcinoma accounted for 21 (10.9%) cases in the non-CGP group but was not observed in the CGP group. The prevalence of most comorbidities was generally comparable between the groups, except for cardiac disease, which was significantly more frequent in the non-CGP group (43 (22.3%) vs. 5 (8.3%), P = 0.016). Figure 1 shows OS in the CGP and non-CGP groups. No significant difference in OS was observed between the groups (median OS: 22.9 months in the CGP group vs. 23.0 months in the non-CGP group, P = 0.78). In addition, OS was evaluated according to the presence or absence of CGP testing within major tumor types, including colorectal, gastric, pancreatic, and biliary tract cancers; no significant differences in OS were observed for any of these cancer types (Figure 2).

**Figure 3:**
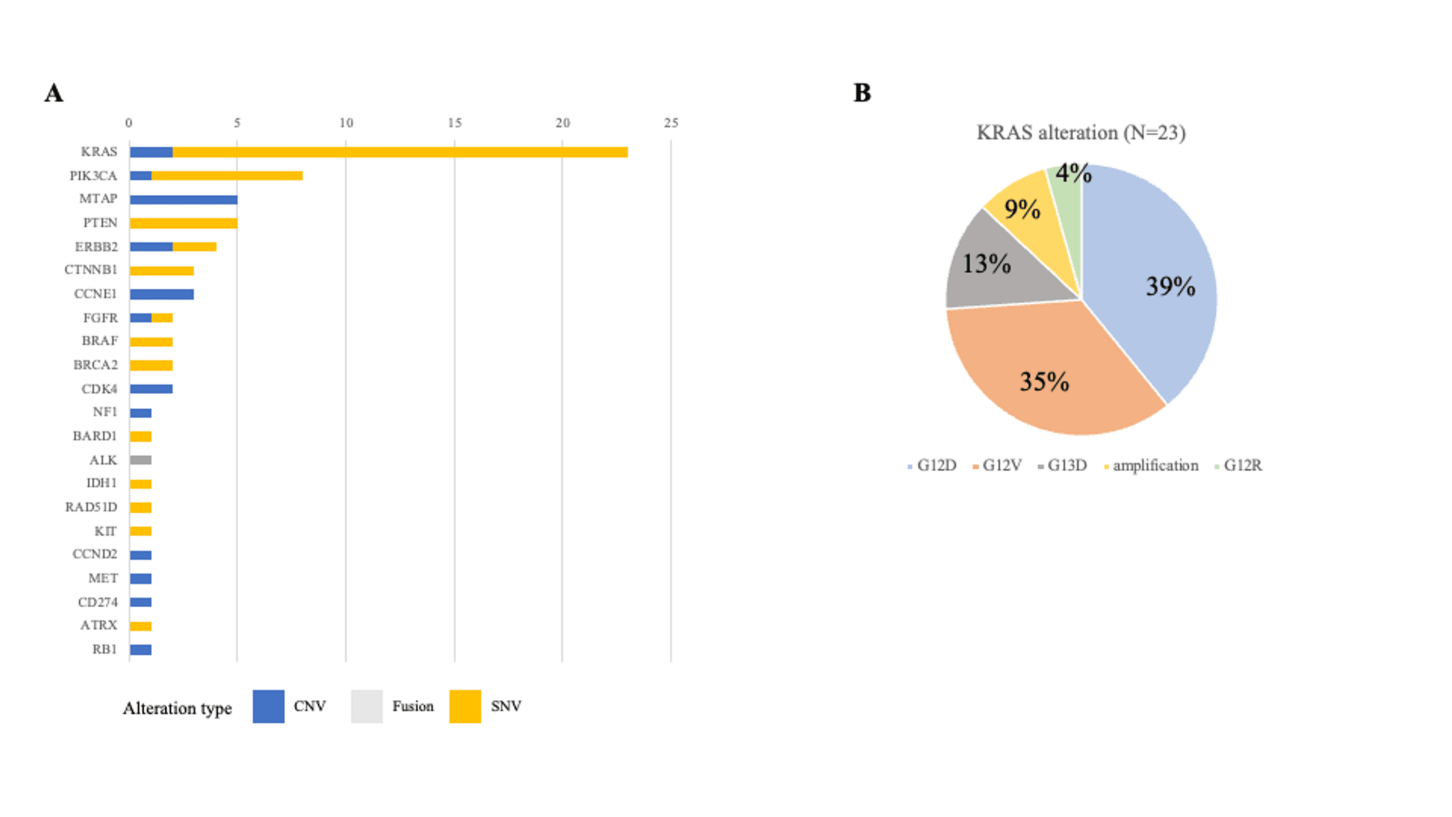
(A) Druggable genomic alterations detected by CGP. (B) KRAS alteration profile in druggable genomic alterations. (A) The frequency of druggable genomic alterations identified in this study. Among the detected alterations, KRAS alterations were the most common. (B) The breakdown of KRAS alterations, with KRAS G12D accounting for nearly 40% of the cases. CGP, comprehensive genomic profiling

**Figure 4:**
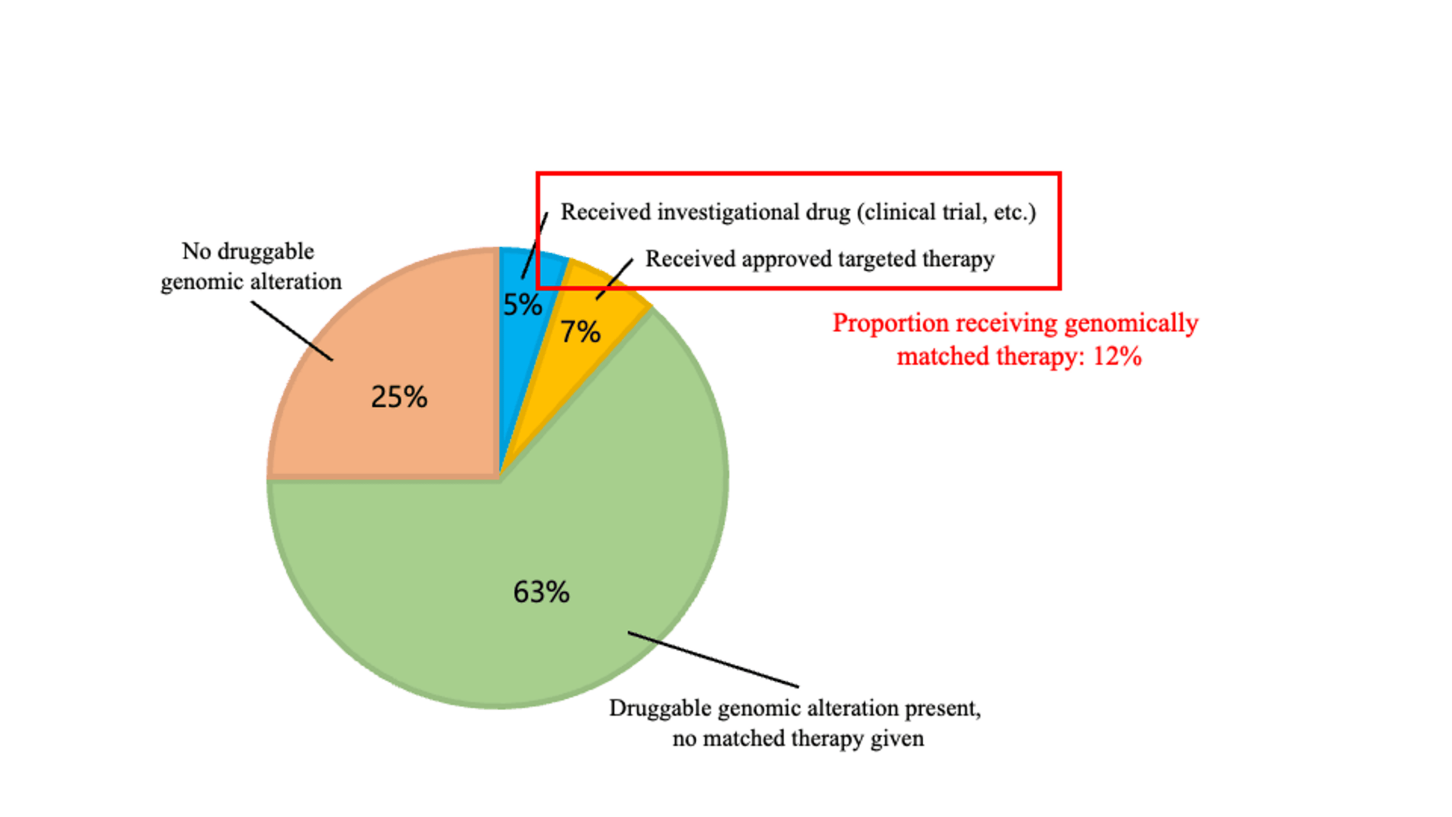
Results of CGP testing. Druggable genomic alterations were identified in 75% of patients who underwent CGP testing in this study. Among them, 12% were able to receive genomically matched therapy. Of those who received such therapy, 5% were treated within clinical trials, while 7% received approved targeted therapy. CGP, comprehensive genomic profiling

Subsequently, the 60 patients who underwent CGP testing were analyzed. Among these 60 patients, druggable alterations were identified in 45 (75.0%) (Table 2). The types and frequencies of druggable alterations observed in this study are shown in Figure 3. KRAS alterations were the most frequent and were mainly detected in patients with pancreatic cancer. In addition, HER2 alterations, PIK3CA alterations, and BRCA alterations were identified, all of which serve as companion diagnostic biomarkers in several tumor types. Genomically matched therapy targeting the detected alterations was administered in 7 (11.7%) of the 60 patients (Figure 4). Of these 7 patients, 3 received investigational agents through clinical trials and 4 received approved agents available in routine clinical care. Details of these cases are summarized in Table 2. In addition, Table 3 summarizes the distribution of druggable genomic alterations and the implementation of genomically matched therapy according to cancer type among patients who underwent CGP testing. Druggable alterations were identified across multiple tumor types, and the proportion of patients who received genomically matched therapy varied by primary site. Table 4 details individual cases in which genomically matched therapy was delivered based on CGP results, including the key alteration(s) supporting treatment selection, access route (clinical trial vs. approved agents used in routine practice), and treatment outcomes. Objective responses were observed in representative cases, and the clinical course and response duration are summarized in Table 4. By contrast, although recommended targeted agents were available in 38 (63.3%) of the 60 patients, genomically matched therapy could not be delivered; the reasons are listed in Table 5. Two representative cases in which genomically matched therapy resulted in an objective response are described below.

**Table 2:** Detailed information of patients received by the CGP test in this study. CGP, comprehensive genomic profiling

Case	Type of cancer	Types of CGP assays	Actionable genomic alteration	Druggable genomic alteration	Genomically matched therapy
1	Colorectal cancer	FoundationOne® CDx	APC	None	None
2	Colorectal cancer	FoundationOne® CDx	APC, MYC amp,	None	None
3	Colorectal cancer	FoundationOne® CDx	APC	None	None
4	Biliary tract cancer	FoundationOne® CDx	NF1 loss	NF1 loss	None
5	Colorectal cancer	FoundationOne® CDx	None	None	None
6	Biliary tract cancer	FoundationOne® Liquid CDx	None	None	None
7	Colorectal cancer	FoundationOne® CDx	APC	None	None
8	Colorectal cancer	FoundationOne® CDx	KRASG12D	KRASG12D	None
9	Breast cancer	FoundationOne® CDx	TMB-H(14Muts/Mb), PIK3CA, BARD1, MYC amp	TMB-H(14Muts/Mb), PIK3CA, BARD1	Pembrolizumab
10	Duodenal cancer	FoundationOne® CDx	ALK EML4 rearrangement	ALK EML4 rearrangement	Alectinib
11	Colorectal cancer	FoundationOne® CDx	APC, GNAS	None	None
12	Colorectal cancer	FoundationOne® CDx	KRASG13D, APC	KRASG13D	None
13	Gastric cancer	FoundationOne® CDx	PIK3CA	PIK3CA	None
14	Breast cancer	FoundationOne® CDx	PIK3CA, PTEN	PIK3CA, PTEN	None
15	Pancreas cancer	FoundationOne® CDx	KRASG12R, MYC amp	KRASG12R	None
16	Endometrial cancer	FoundationOne® CDx	PTEN	PTEN	None
17	Colorectal cancer	FoundationOne® CDx	KRASG13D, APC	KRASG13D	None
18	Esophageal cancer	FoundationOne® CDx	MTAP loss, CTNNB1	MTAP loss, CTNNB1	None
19	Biliary tract cancer	FoundationOne® CDx	IDH1	IDH1	None
20	Gastric cancer	FoundationOne® CDx	BRAF L485W, MTAP loss, KRAS amp	BRAF L485W, MTAP loss, KRAS amp	None
21	Colorectal cancer	FoundationOne® CDx	APC, FLT3	None	None
22	Gastric cancer	FoundationOne® CDx	FGFR4	FGFR4	None
23	Biliary tract cancer	FoundationOne® CDx	ERBB2 S310F, APC	ERBB2 S310F	None
24	Biliary tract cancer	FoundationOne® CDx	KRASG12D, MTAP loss, RAD51D	KRASG12D, MTAP loss, RAD51D	None
25	Pancreas cancer	FoundationOne® CDx	KRASG12V, MTAP loss, PIK3CA amp, CDKN2A/B	KRASG12V, MTAP loss	None
26	Colorectal cancer	FoundationOne® CDx	KRASG12D, APC, PIK3CA	KRASG12D, PIK3CA	None
27	Breast cancer	FoundationOne® CDx	PIK3CA	PIK3CA	Capivasertib
28	Gastric cancer	FoundationOne® CDx	MYC amp, FLT3, BCL2L1	None	None
29	Biliary tract cancer	FoundationOne® CDx	KRASG12V, CDKN2A/B	KRASG12V	None
30	Colorectal cancer	FoundationOne® CDx	APC	None	None
31	Pancreas cancer	FoundationOne® CDx	KRASG12D, BRAF H574Q	KRASG12D, BRAF H574Q	None
32	Biliary tract cancer	FoundationOne® CDx	BRCA2, CDKN2A/B, CTNNB1	BRCA2, CTNNB1	None
33	Biliary tract cancer	FoundationOne® CDx	TMB-H(35), ERBB2 amp, CDK4 amp	TMB-H(35), ERBB2 amp, CDK4 amp	Pembrolizumab
34	Colorectal cancer	FoundationOne® CDx	KRASG12V, APC, AURKA amp	KRASG12V,	None
35	Biliary tract cancer	FoundationOne® CDx	KRASG12V, FGFR1 amp, MYC amp	KRASG12V	None
36	Gastric cancer	FoundationOne® CDx	ATM, CCND1	None	None
37	Biliary tract cancer	FoundationOne® CDx	KRASG12V, BRCA2	KRASG12V, BRCA2	None
38	Biliary tract cancer	FoundationOne® CDx	MSI-H (MSH6), TMB-H (34), ATM, CTNNB1, ERBB3	MSI-H (MSH6), TMB-H (34), CTNNB1	Pembrolizumab
39	Ovarian cancer	FoundationOne® CDx	CCNE1 amp, PIK3CA, KIT	CCNE1 amp, PIK3CA, KIT	None
40	Biliary tract cancer	FoundationOne® CDx	CCND2, PTEN	CCND2, PTEN	None
41	Colorectal cancer	FoundationOne® CDx	TMB-H (10), MET amp, CD274 amp	TMB-H (10), MET amp, CD274 amp	ABBV400
42	Biliary tract cancer	FoundationOne® CDx	KRAS amp	KRAS amp	None
43	Pancreas cancer	FoundationOne® CDx	KRASG12D, CDKN2A/B	KRASG12D	None
44	Ovarian cancer	FoundationOne® CDx	None	None	None
45	Biliary tract cancer	FoundationOne® CDx	ERBB2 S310Y, APC, CDKN2A/B, PBRM1	ERBB2 S310Y	None
46	Pancreas cancer	FoundationOne® CDx	KRASG12D, CDKN2A, CCNE1 amp	KRASG12D, CCNE1 amp	None
47	Pancreas cancer	FoundationOne® CDx	KRASG12V, MTAP loss, CDKN2A/B loss,	KRASG12V, MTAP loss	None
48	Colorectal cancer	FoundationOne® CDx	APC	None	None
49	Pancreas cancer	FoundationOne® CDx	KRASG12D, CDKN2A/B, CCND3 amp	KRASG12D,	None
50	Biliary tract cancer	FoundationOne® CDx	MYC amp, CDKN2A	None	None
51	Colorectal cancer	FoundationOne® CDx	APC, NRASG12D	None	None
52	Biliary tract cancer	FoundationOne® CDx	PTEN, CDKN2A/B	PTEN	None
53	Colorectal cancer	FoundationOne® CDx	APC, KRASG13D	KRASG13D	None
54	Pancreas cancer	FoundationOne® CDx	KRASG12D, CCNE1 amp	KRASG12D, CCNE1 amp	None
55	Pancreas cancer	FoundationOne® CDx	KRASG12V, CDKN2A,	KRASG12V	None
56	Pancreas cancer	FoundationOne® CDx	KRASG12V, AKT2 amp, CDKN2A/B loss	KRASG12V	None
57	Pancreas cancer	FoundationOne® CDx	KRASG12D, CCND1 amp, CDKN2A	KRASG12D, CCND1 amp	None
58	Retroperitoneal liposarcoma	GenMineTOP	CDK4 amp, ATRX	CDK4 amp, ATRX	None
59	Biliary tract cancer	FoundationOne® CDx	RB1 loss, PIK3CA, PTEN	RB1 loss, PIK3CA, PTEN	None
60	Biliary tract cancer	FoundationOne® CDx	ERBB2 amp, CDKN2A	ERBB2 amp	TDxd

**Table 3:** Druggable alterations and genomically matched therapy by tumor type in the CGP cohort (n = 60). “Druggable alterations” were defined as genomic alterations judged to be treatable by the molecular tumor board, including approved agents, investigational drugs, and off-label use when applicable. “Genomically matched therapy” was defined as treatment actually administered based on CGP results. CGP, comprehensive genomic profiling

Cancer types	N	Druggable alterations, n (%)	Genomically matched therapy, n (%)
Biliary tract cancer	18	16 (88.9)	3 (16.7)
Colorectal cancer	17	7 (41.1)	1 (5.9)
Pancreatic cancer	11	11 (100.0)	0 (0.0)
Gastric cancer	5	3 (60.0)	0 (0.0)
Breast cancer	3	3 (100.0)	2 (66.7)
Ovarian cancer	2	1 (50.0)	0 (0.0)
Esophageal cancer	1	1 (100.0)	0 (0.0)
Endometrial cancer	1	1 (100.0)	0 (0.0)
Duodenal cancer	1	1 (100.0)	1 (100.0)
Retroperitoneal liposarcoma	1	1 (100.0)	0 (0.0)

**Table 4:** Rationale for genomically matched therapy and treatment summary (n = 7). The “Key alteration(s)” column summarizes the primary biomarker(s) that provided the biological/clinical rationale for selecting the matched therapy (e.g., ALK fusion → ALK inhibitor; TMB-high/MSI-high → immune checkpoint inhibitor; ERBB2 amplification → HER2-targeted therapy; MET amplification → MET-targeted investigational agent). ADC, antibody drug conjugate; CR, complete response; MSI, microsatellite instability; PD, progressive disease; PR, partial response; SD, stable disease; TMB, tumor mutation burden

Case no.	Cancer types	Key alteration(s) supporting the chosen therapy	Genomically matched therapy	Access route	Best response
9	Breast cancer	TMB-High	Pembrolizumab	Approved (tumor-agnostic for TMB-H/MSI-H)	PD
10	Duodenal cancer	ALK EML4 rearrangement	Alectinib	Clinical trial	PR
27	Breast cancer	PIK3CA mutation	Capivasertib	Clinical trial	SD
33	Biliary tract cancer	TMB-High	Pembrolizumab	Approved (tumor-agnostic for TMB-H/MSI-H)	CR
38	Biliary tract cancer	MSI-high and TMB-high	Pembrolizumab	Approved (tumor-agnostic for TMB-H/MSI-H)	CR
41	Colorectal cancer	MET amplification	ABBV400 (c-Met-targeting ADC)	Clinical trial	PD
60	Biliary tract cancer	ERBB2 amplification	Trastuzumab deruxtecan	Clinical trial	PR

**Table 5:** Reasons for not enrolling in genomically matched clinical trials among patients with druggable alterations identified by CGP (n = 38). CGP, comprehensive genomic profiling

Reason category	n (%)
The patient’s clinical condition precluded referral to a designated cancer genomic medicine core hospital.	18 (47.4)
The patient visited a designated cancer genomic medicine core hospital but could not be enrolled in a clinical trial for various reasons (e.g., trial closure, clinical deterioration while on the waiting list, exclusion because of comorbidities).	11 (28.9)
The patient and their family declined referral to a designated cancer genomic medicine core hospital.	9 (23.7)

**Figure 5:**
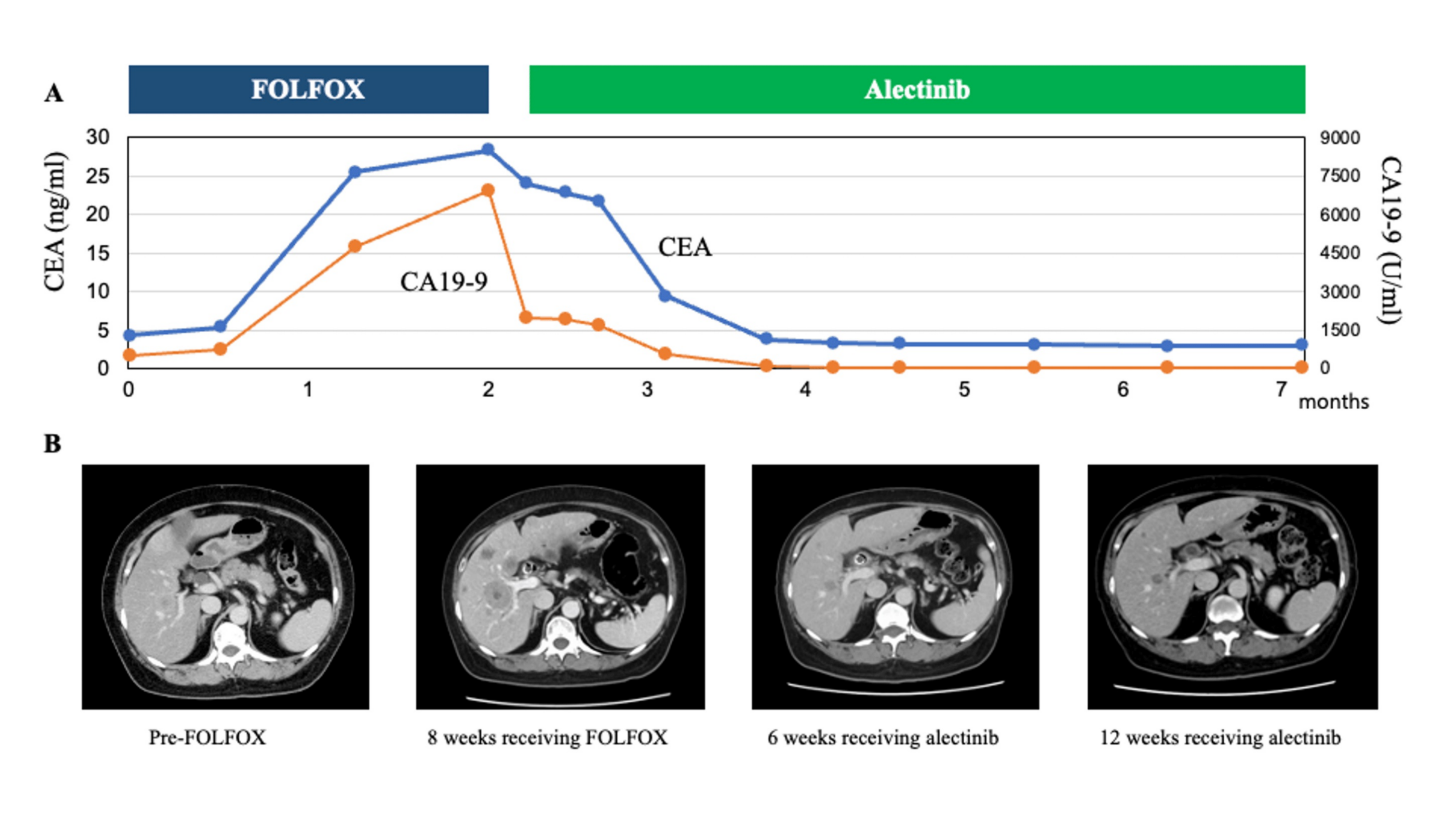
Timeline of case 10 (a patient with ALK-fusion duodenal carcinoma). (A) Changes in tumor marker levels (carcinoembryonic antigen (CEA) and carbohydrate antigen 19-9 (CA 19-9)) during treatment with FOLFOX followed by alectinib. (B) Abdominal computed tomography demonstrating multiple liver metastases before FOLFOX therapy, increased tumor burden four weeks after FOLFOX, and reduction of liver metastases six weeks after initiating alectinib. The second follow-up CT scan showed a continued response of the liver metastases.

The first case was a 62-year-old woman with duodenal cancer and liver metastases (case 10). Modified FOLFOX6 was initiated as first-line chemotherapy, and CGP using a tissue specimen was performed in parallel. At the time of progression on first-line therapy, CGP revealed an ALK-EML4 rearrangement. The patient was subsequently enrolled in a clinical trial (BELIEVE study: NCCH1901, jRCTs031190104), and treatment with alectinib was initiated. The best overall response was a partial response, and disease control was maintained for almost two years. Details of this case are presented in Figure 5.

**Figure 6:**
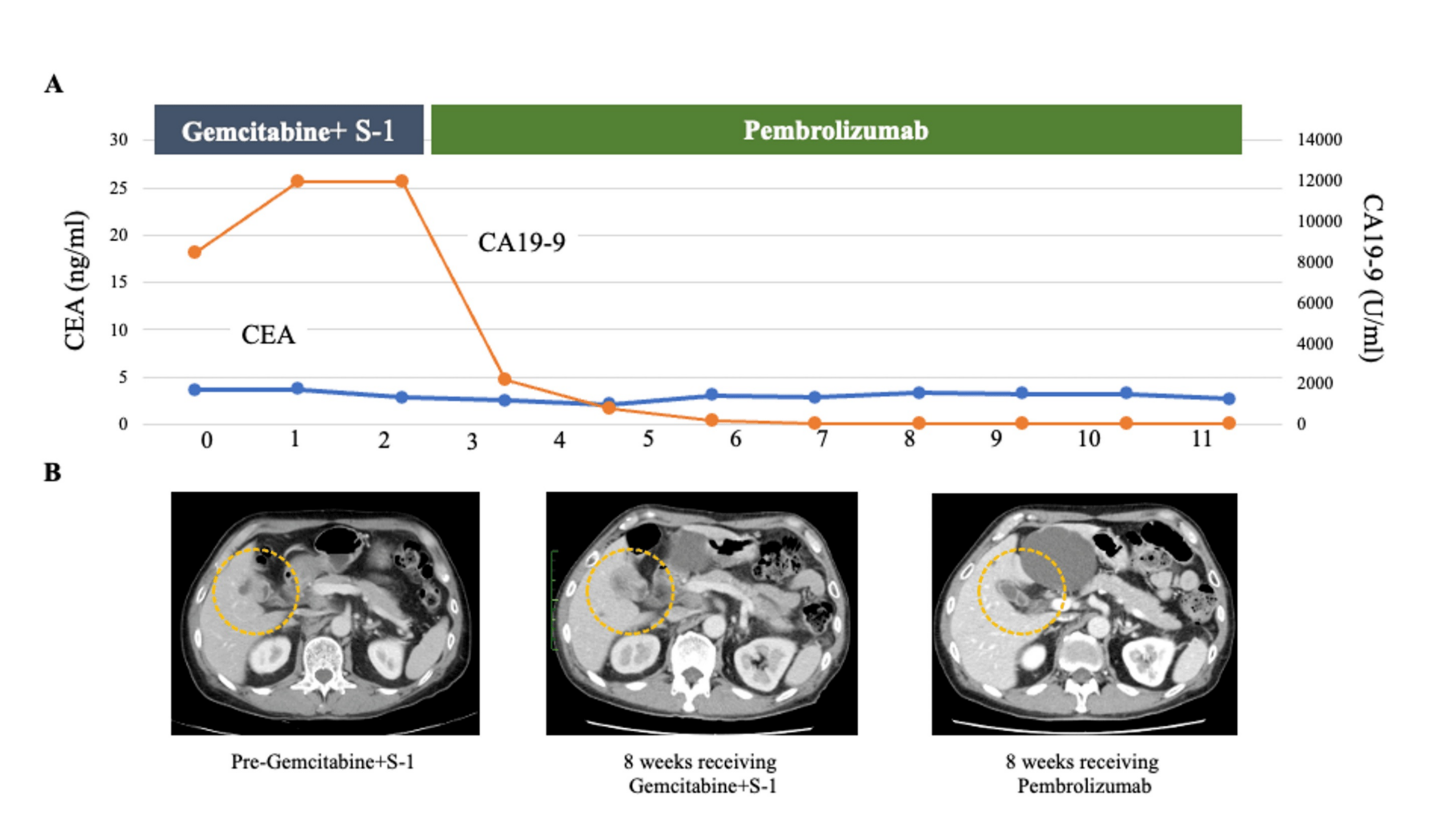
Timeline of case 33 (a patient with high tumor mutational burden (TMB) gallbladder carcinoma). (A) Changes in tumor marker levels (CEA and CA 19-9) while receiving treatment with gemcitabine plus S-1 and pembrolizumab. (B) Abdominal computed tomography demonstrating gallbladder carcinoma before gemcitabine plus S-1 therapy, increased tumor burden eight weeks after gemcitabine plus S-1 therapy, and reduction of liver metastases eight weeks after initiating pembrolizumab.

The second case was a 67-year-old man with gallbladder carcinoma and liver metastases (case 33). First-line gemcitabine plus S-1 (GS) chemotherapy was initiated, and CGP testing using a tissue specimen was performed in parallel. After two months, the disease progressed on GS therapy. CGP revealed a TMB-high status (35 mutations/Mb) and ERBB2 amplification. Although the patient could not be enrolled in a clinical trial targeting ERBB2 amplification, pembrolizumab, which is approved for tumors with TMB-high status, was started. The best overall response was a complete response, and more than two years after initiation of pembrolizumab, the patient remains progression-free. Details of this case are illustrated in Figure 6.

**Figure 7:**
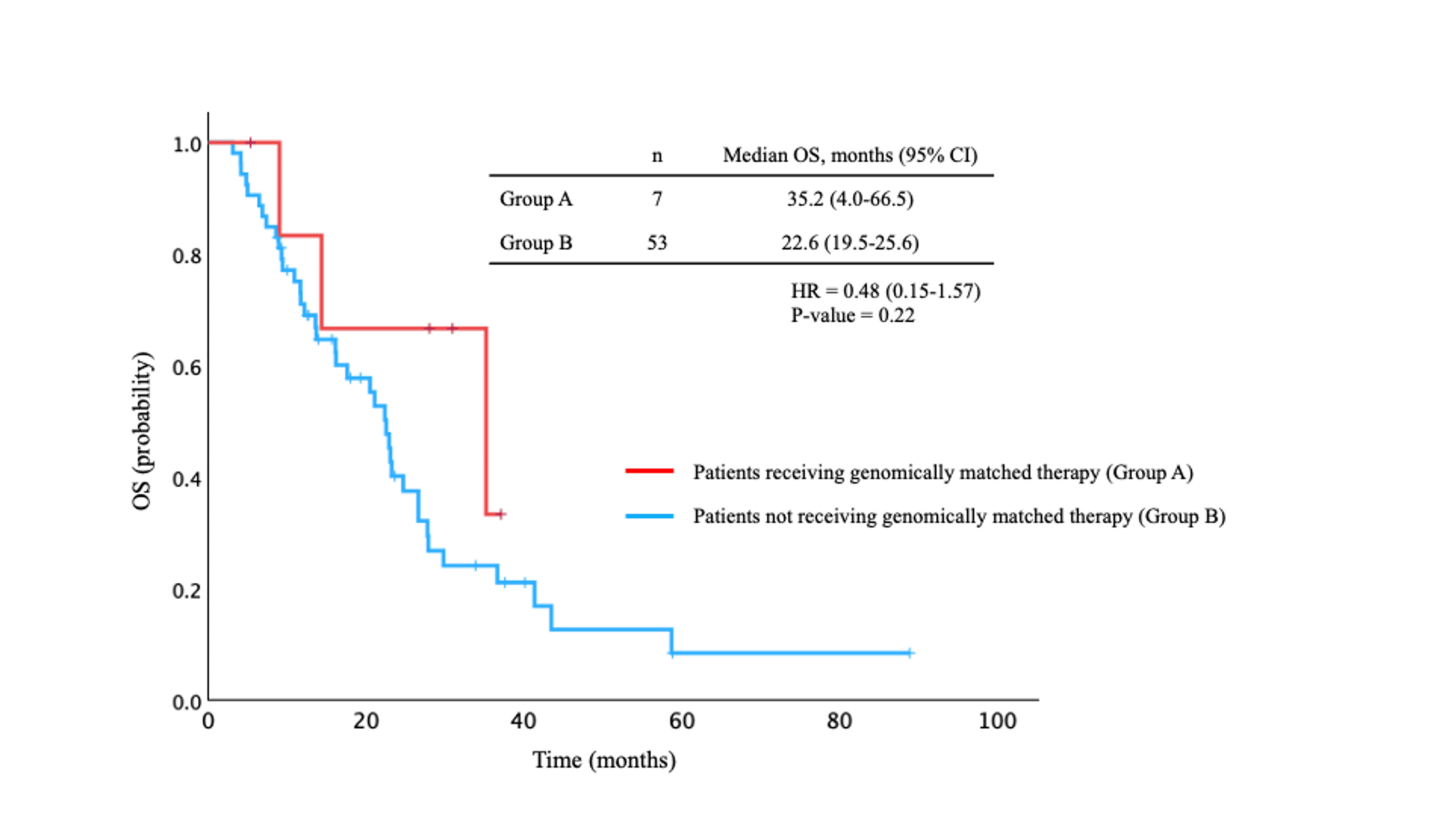
Overall survival (OS) curves in the comprehensive genomic profiling (CGP) groups. Median OS was 35.2 months (95% CI, 4.0-66.5 months) in the matched group and 22.6 months (95% CI, 19.5-25.6 months) in the non-matched group; however, this difference was not statistically significant (HR 0.48, 95% CI 0.15-1.57; P = 0.22).

Among the 60 patients who underwent CGP, OS was also evaluated between those who received genomically matched therapy and those who did not (Figure 7). Median OS was 35.2 months (95% CI, 4.0-66.5 months) in the matched group and 22.6 months (95% CI, 19.5-25.6 months) in the non-matched group; however, this difference was not statistically significant (HR 0.48, 95% CI 0.15-1.57; P = 0.22).

## Discussion

In this study, the clinical utility of CGP in a community hospital setting was evaluated. As a result, no difference in OS was observed according to whether CGP testing was performed. On the other hand, more than 10% of patients received genomically matched therapy based on “druggable genomic alterations” identified by CGP, and this proportion was comparable to that reported from designated core hospitals for cancer genomic medicine [9,11]. These findings suggest that, even in general community hospitals where CGP testing itself is not available on site, CGP can still provide meaningful benefits for patients with cancer when appropriate case selection and collaborative referral pathways are established. Importantly, the clinical impact of CGP is not expected to be uniform across all tumor types, because the spectrum of genomic alterations and the real-world accessibility of effective genotype-matched therapies differ substantially by primary site. Thus, CGP utility should be interpreted not only through OS in an unselected cohort, but also through tumor-type-specific deliverability of matched options and the clinical context in which CGP is used.

The clinical utility of CGP has been demonstrated in several large‐scale cohorts, including MSK‐IMPACT2, NCI‐MATCH, IPREDICT, and SCRUM‐Japan, in which a certain proportion of patients were found to harbor actionable or druggable genomic alterations, and a subset of these patients ultimately received genome-driven therapies such as molecularly targeted agents or immune checkpoint inhibitors [11,12]. However, many reports have shown that, despite a relatively high detection rate of druggable alterations, the proportion of patients who actually receive matched therapy remains low, particularly in gastrointestinal cancers, where this rate is reported to be approximately 10-20% [8,9]. The matched-therapy rate observed in the present study (over 10%) falls within this previously reported range, suggesting that precision oncology can be delivered in community hospitals at a level comparable to that of high-volume centers. This observation also highlights the well-recognized “implementation gap” between identifying potentially druggable alterations and successfully delivering genotype-matched therapy in routine practice, which is shaped by the evidence level for alteration-drug pairs and access to corresponding treatments.

By contrast, the present study did not demonstrate a difference in OS according to whether CGP testing was performed. Several factors may account for this finding. The most important is the marked difference in tumor types between the two groups. The CGP group included a higher proportion of poor-prognosis malignancies, such as pancreatic cancer, biliary tract cancer, and various rare cancers, whereas the non-CGP group contained more tumor types with relatively favorable expected outcomes, including breast cancer and hepatocellular carcinoma. Given that CGP testing is often ordered with the additional aim of expanding treatment options for patients with limited therapeutic choices, the population undergoing CGP tends to be enriched for diseases with an intrinsically poor prognosis.

Second, in Japan, access to clinical trials, investigator-initiated trials, and off-label use remains limited, and there are many cases in which patients harboring druggable alterations are not actually able to receive matched therapies [8,9]. In the present cohort as well, a substantial number of patients could not reach genome-driven therapy because of system-level factors such as trial closure, restricted enrollment slots, clinical deterioration while awaiting trial registration, or ineligibility due to comorbidities. Moreover, the likelihood of translating a detected alteration into treatment differs by alteration class: tumors dominated by alterations with limited direct druggability (e.g., common tumor suppressor losses) may yield fewer actionable pathways than those enriched for therapeutically tractable drivers (e.g., selected kinase fusions or high-evidence oncogenic alterations). Another important consideration is the biology of advanced-stage disease. As cancers progress, mutational burden and clonal diversity often increase through ongoing clonal evolution, resulting in pronounced spatial and temporal intratumoral heterogeneity [13]. Consequently, a single tissue biopsy obtained at one time point may not capture all clinically relevant subclones across multiple metastatic deposits, and resistant subclones that are not represented in the sampled lesion may drive early treatment failure despite the presence of a “druggable” alteration [14]. These findings are consistent with previous reports indicating that CGP testing itself is not an intervention that directly prolongs survival, but rather a diagnostic tool that supports therapeutic decision-making [8,15-17]. To more accurately assess its impact on OS, prospective interventional studies and appropriately matched comparative analyses will be required. In addition, reporting tumor-type-stratified matched-therapy deliverability and the rationale for therapy selection may provide a more clinically informative framework for interpreting CGP utility than an OS comparison alone.

Another notable feature of this study is that more than 20% of patients wished to undergo CGP testing but did not wish to be referred to high-volume centers, such as cancer centers or university hospitals, where clinical trials are available. Many of these patients were elderly and lived near our hospital. For such patients, in whom druggable genomic alterations are identified but participation in clinical trials is difficult, a healthcare system that allows access to genotype-guided therapies within routine insurance-covered practice, irrespective of tumor type, would be desirable. In routine practice, genotype-matched treatment prioritization is largely driven by (i) alterations linked to approved standard therapies within or across tumor types and (ii) alterations enabling enrollment in clinical trials; therefore, treatment deliverability depends not only on detection but also on the evidence level supporting each alteration-drug pair and practical accessibility to the corresponding therapy. In Japan, pembrolizumab for TMB-high tumors, entrectinib and larotrectinib for tumors harboring NTRK fusions, and dabrafenib plus trametinib for tumors with BRAF mutations are already approved for use across solid tumors based on CGP results, regardless of the primary cancer site [18-21]. Although pembrolizumab was selected based on a TMB-high result, TMB is an imperfect predictive biomarker [22]. TMB-high is thought to reflect increased tumor immunogenicity and neoantigen burden; however, it does not directly capture key determinants of immune response such as PD-1/PD-L1 signaling, tumor-infiltrating lymphocyte density, antigen presentation competency, or T-cell functional states (e.g., exhaustion). Accordingly, a TMB-high status does not uniformly translate into clinical benefit, and predictive performance may vary across tumor types, including biliary tract cancers. In the present case, pembrolizumab was initiated because TMB-high is an approved tumor-agnostic biomarker, and alternative effective options were limited; the durable response observed highlights that TMB-high can identify a subset of patients who may benefit, while emphasizing the need for more refined immune-biomarker integration [18]. In addition, trastuzumab deruxtecan, an antibody-drug conjugate, has demonstrated antitumor activity in HER2-mutant solid tumors and may be approved in the future [23]. In the present cohort as well, several cases with HER2 mutations were detected. As the number of molecularly targeted agents that can be administered as reimbursed standard care based on CGP findings continues to increase, the rate of genomically matched therapy in community hospitals can be expected to improve. Notably, frequent alterations are not necessarily synonymous with high deliverability of matched therapy; for example, KRAS alterations were common in this cohort (primarily in pancreatic cancer), yet effective matched strategies for several KRAS variants remain limited and often depend on clinical trial availability, which may further widen the gap between detection and treatment.

Despite the above-mentioned limitations in community hospitals, the implementation of CGP testing in regional settings still appears to have considerable value. First, CGP may contribute to elucidating disease etiology and identifying hereditary tumors in patients with rare cancers, early-onset cancers, or a relevant family history [24,25]. Second, even when genome-matched therapy cannot be provided at this hospital, CGP testing can function as a “gateway” to broaden treatment opportunities through referral to cancer genomic core hospitals or clinical trial sites. Third, by providing information on mechanisms of treatment resistance and prognostic factors, CGP testing may facilitate prognostic counseling for patients and their families, and support shared decision-making regarding subsequent care plans, including transition to best supportive care.

This study has several limitations. First, it was a single-center retrospective study with a limited number of patients; therefore, the generalizability of the findings should be interpreted with caution. Second, substantial selection bias is likely to exist between the CGP and non-CGP groups in terms of tumor type, treatment history, and PS, and residual confounding in the comparison of OS cannot be excluded. Third, because a wide variety of tumor types were included, the number of cases was insufficient to evaluate in detail the utility of CGP for any specific cancer type. Fourth, although the institution is a community hospital, it is favorably located, and patients have relatively easy access to cancer genomic core hospitals. In contrast, patients treated at hospitals in rural areas may need to travel several hours to undergo CGP testing. Whether CGP testing truly confers clinical benefit for patients treated at such facilities requires separate investigation. Despite these limitations, the present study provides real-world data on the current status, challenges, and feasibility of CGP testing in Japanese community hospitals and may offer useful insights for future development of genomic medicine infrastructures and for determining the optimal timing of CGP implementation.

## Conclusions

Although this study was a small, retrospective analysis, it demonstrated that CGP testing in a community hospital setting was feasible and enabled a clinically meaningful proportion of patients to receive genome-matched therapy. The proportion of patients who received genomically matched therapy in this cohort (7/60, 12%) was comparable to rates reported in prior Japanese real-world studies from designated cancer genomic medicine hospitals/core centers. Many areas for improvement remain in order to increase the proportion of patients who can receive genome-matched therapy; however, the present study indicates that CGP testing in community hospital settings is by no means futile. CGP in community hospitals is feasible and yields clinically meaningful opportunities for genome-matched therapy.
